# The Power of Birth Cohorts to Study Risk Factors for Cognitive Impairment

**DOI:** 10.1007/s11910-022-01244-0

**Published:** 2022-11-09

**Authors:** Marcus Richards

**Affiliations:** MRC Unit for Lifelong Health and Ageing at UCL, University College London, 1-19 Torrington Place, London, WC1E 7HB UK

**Keywords:** Birth cohort, Life course, Cognition, Cognitive impairment, Dementia

## Abstract

**Purpose of Review:**

Birth cohorts are studies of people the same time; some of which have continuously followed participants across the life course. These are powerful designs for studying predictors of age-related outcomes, especially when information on predictors is collected before these outcomes are known. This article reviews recent findings from these cohorts for the outcomes of cognitive function, cognitive impairment, and risk of dementia, in relation to prior cognitive function, and social and biological predictors.

**Recent Findings:**

Cognitive function and impairment are predicted by a wide range of factors, including childhood cognition, education, occupational status and complexity, and biological factors, including genetic and epigenetic. The particular importance of high and rising blood pressure in midlife is highlighted, with some insight into brain mechanisms involved. Some limitations are noted, including sources of bias in the data.

**Summary:**

Despite these limitations, birth cohorts have provided valuable insights into factors across the life course associated with cognitive impairment.

## Introduction

This chapter reviews how birth cohorts inform studies of cognitive ageing, cognitive impairment, and risk of dementia. Thus, the focus is on the contribution of factors across the life course. As will shortly be discussed, there are several methods for obtaining such information, from recruiting a cohort at birth and continuously following it, to recruiting later in life then linking back to historical records. This chapter cannot possibly present an exhaustive review of findings across all birth cohorts, which would require a book. Rather, some key topics have been selected, mostly based on recent findings: life course models of cognitive ageing, social influences, and the biological influences of genetics and cardiovascular function. For convenience, these topics are presented separately but of course are closely interrelated in life. For related findings presented in chronological order over the life course, the reader is referred to Richards and Deary [1].

## What Is a Birth Cohort?

Within the human sciences, a cohort is defined as a group of people with a shared characteristic. In a birth cohort, this characteristic is being born approximately the same time. This window can be very narrow, for example 1 week of the same year, or spanning a decade or more. Thus, by design differences in outcomes at any life stage of a birth cohort are due to factors other than chronological age, although obviously the narrower the age range of the cohort the easier age effects per se can be ruled out. Most research using these cohorts attempts to identify these influencing factors, including genes, environmental exposures and individual behaviours, and their separate and combined effects on functions and their trajectories. Birth cohorts born in different eras, or at approximately the same age but in different regions, can also be compared, to investigate whether outcomes are affected by external differences, for example in healthcare or societal structures such as schooling and the workplace. When outcomes are compared at the same age across different cohorts, any difference is referred to as a cohort effect. For example, in several cultures left-handedness has become more prevalent in children of younger generations, due to increasing societal acceptance and corresponding decrease in forced change. In contrast, a period effect results from a something affecting all ages simultaneously, although not necessarily to the same degree. At the time of writing, the COVID-19 pandemic is a striking example, since this can affect most people over a short period, although often with more severe outcomes in older people. Most birth cohorts are observational, with no attempt by investigators to actively manipulate exposures. These cohorts are mainly prospective, ideally observing exposures and outcomes as they occur. However, the often-lengthy gaps between assessments introduce the necessity of recall, with associated risks of error and bias.

## Examples of Birth Cohorts and Their Designs

Since the focus of this chapter is on cognition, the emphasis is on findings from birth cohorts with relevant outcomes in later life or approaching this. Some of these cohorts have been continuously followed across the life course. One of the oldest is the Wisconsin Longitudinal Study (WLS), which has followed participants to an age where dementia can be ascertained [2]. WLS is based on a one-in-three random sample of Wisconsin high school graduates aged around 20 years, born between 1938 and 1940 (*n* = 10,317) and continually assessed, most recently in 2020 around age 83 years. At age 16 years, participants were administered Henmon-Nelson tests of cognitive ability, which include scores for verbal, numerical, and visuospatial ability. Impossible to estimate retrospectively, these test scores are invaluable as predictors of outcomes, including later life cognition.

The MRC National Survey of Health and Development (NSHD; the 1946 birth cohort) initially consisted of 5362 males and females born in a single week of that year in mainland Britain and continuously followed to current age 76 [3, 4]. At ages 8, 11, and 15 years, participants were administered tests of verbal and nonverbal cognitive ability. NSHD also contains Insight 46, a neuroscience sub-study, originally consisting of 502 quasi-randomly selected participants [5, 6]. These participants undergo simultaneous positron emission tomography (PET) for amyloid deposition and magnetic resonance imaging (MRI) for high resolution volumetric scans, and other sequences including resting state functional MRI; diffusion-weighted MRI; and arterial spin labelling for quantitative mapping of cerebral blood flow.

The National Child Development Study (NCDS; the 1958 birth cohort) [7] initially consisted of 17,415 males and females born in mainland Britain during a single week of that year. Verbal and nonverbal cognitive ability were assessed in childhood at ages close to those of NSHD. At the time of writing, the latest round of assessments in this cohort is in progress, around age 62, including tests of fluid cognitive function. This is of course still relatively young for age-associated cognitive impairment but will provide the baseline for its future tracking. The most recent completed cognitive tests were administered at age 50, and with this caveat in mind, findings from these are considered below.

That caveat also applies to the Dunedin Multidisciplinary Health and Development Study, which recruited nearly all infants at birth in one hospital between April 1972 and March 1973, 1037 (91%) of who were followed up at age 3 years [8]. Now disbursed around the world, these participants are flown back to Dunedin for assessments, ensuring an unusually high retention rate. Full-scale IQ was measured at ages 7, 9, and 11 years, then again at age 45 along with a comprehensive neuropsychological test battery.

Despite the name, a birth cohort does not have to begin data collection at birth. Some were recruited in later life to link back to archived data collected in a different context. In the UK, participants in the Lothian birth cohorts of 1921 and 1936, and the Aberdeen birth cohorts of the same years, were reconstructed in later life from the Scottish Mental Surveys, which tested cognitive ability in almost all 11-year-olds in Scotland during 1 month of those years [9–11]. It was also possible for these studies to link back to obstetric outcomes, such as birthweight, from clinical records. Some studies use date of birth as a proxy for birth cohort without reference to prior data. Using electoral and primary care records, the Caerphilly Prospective Study (CAPS) recruited 2512 men aged 45 to 59 years from the eponymous town and adjoining villages in Wales, UK [12]. Although cognitive function was not assessed at baseline, this was added at phase III, 10 years later, and at phase IV, 4 years after that. Another example is the North America–based Alzheimer’s Disease Neuroimaging Initiative (ADNI), which defined four birth cohorts within its sample, born 1915–1928, 1929–1938, 1939–1945, and 1946–196. The younger the cohort, the higher were scores for fluid cognitive function [13].

## Key Recent Birth Cohort Findings

### Paths to Late Life Cognitive Function and Prediction of Cognitive Impairment

Using path analysis in NSHD, six life course variables from NSHD were linked to the Addenbrooke’s Cognitive Examination (ACE-III, a comprehensive test of cognitive state) at age 69: APOE4, parental social class represented by mother’s education and father’s occupation, cognitive ability at age 8, educational attainment by age 26, and midlife occupational complexity and the National Adult Reading Test (NART) at age 53, a test of crystallised verbal ability. Since path analysis is a variety of regression, all path coefficients were mutually independent. The strongest path to the ACE-III was through cognition itself, from childhood cognition via the NART. This was followed in strength by childhood cognition via education, and to a lesser extent via midlife occupational complexity [14]. There were no direct paths from the parental variables to the ACE-III, although these were positively associated with childhood cognition, education, and midlife occupation, in descending order of strength. APOE4 was directly, negatively and weakly associated with the ACE-III, although not with any of the mediating variables. Similar findings were reported from WLS for late life cognition [15, 16].

A range of independent outcomes was then compared in those whose ACE-III scores were expected, worse and better than predicted from the path model [17••]. Compared with the expected group, those in the worse group showed faster memory decline, more self-reported memory difficulties, more difficulties with instrumental activities of daily living, greater likelihood of being independently rated by experienced specialist clinicians as having a progressive cognitive impairment, and a cortical thinning pattern suggestive of preclinical Alzheimer’s disease. In sum, those who performed worse on the ACE-III than predicted by the above path model showed independent evidence suggesting increasing risk of dementia.

### Links Between Depression and Cognition

Depression is a well-known risk factor for dementia. In the Livingston Dementia Prevention, Intervention, and Care report [18], later life depression accounts for 4% of potentially modifiable risk of this outcome. In CAPS, Spielberger Trait Anxiety was associated with elevated risk of cognitive impairment and dementia 17 years later, controlling for age, vascular risk factors, and premorbid cognition [12]. In fact, emotional symptoms and cognitive function are entwined across the life course, and a large effort has been spent clarifying directionality and common cause. Although childhood cognition predicted adult emotional symptoms in NSHD [19], persistence and accumulation of emotional symptoms across adulthood was also inversely associated with subsequent cognition, in NSHD [20–22] and NCDS [23]. This impression of symptom-to-cognition directionality was reinforced by a cross-lagged analysis in NSHD, showed that emotional symptoms predicted poorer verbal memory and processing speed over a period of 16 years, but not vice versa [24••]. In Lothian 1936, mild cognitive impairment (MCI) status was derived using NIA-AA diagnostic guidelines; 14% had MCI at 76 years and 19% at 82 years. Over the follow-up period, 74% remained cognitively healthy, 12% transitioned to MCI, 7% reverted to healthy cognition, and 7% maintained their baseline MCI status. One predictor of group membership was number of depressive symptoms [25•].

Regarding common cause, it is important to note that the depression-cognition association is not specific. In the Dunedin study, participants with emotional disorders not only experienced cognitive difficulties but showed faster pace of biological ageing (indexed by biomarkers of cardiovascular, metabolic, pulmonary, kidney, immune, and dental systems), more difficulties with hearing, balance, and motor functioning, and were rated as looking older [8]. This is consistent with findings in NCDS, where the association between emotional symptoms and subsequent cognition was partly explained by an index of cardiometabolic risk (total and HDL cholesterol, triglycerides, glycosylated haemoglobin, systolic and diastolic blood pressure, C-reactive protein, fibrinogen, and resting heart rate) [26].

We mentioned that emotional symptoms and cognitive function are entwined across the life course and have argued elsewhere that these gradually fuse to form skills for life [27]. These skills, sometimes referred to as ‘non-cognitive’, underpin self-regulation, i.e., ‘self-generated thoughts, feelings, and actions that are planned and cyclically adapted to the attainment of personal goals.’ [28]. This has important implications for cognitive ageing. Indeed, a variable representing self-organisation in NSHD was extracted from teacher ratings when participants were adolescents, and was found to predict memory in midlife, after controlling for adolescent cognitive function [29]. It also predicts body weight over the life course in this cohort [30], and chronic elevation of and rapid rise in waist circumference is itself associated with subsequent cognitive function, controlling for cardiovascular risk factors, physical activity, education, childhood cognition, and socioeconomic position [31].

Some insight into brain anatomy behind inter-relationships between emotional symptoms and cognitive function has been provided by Koike et al. [32]. After mutual adjustment, lower childhood verbal ability in NSHD and greater verbal developmental lag were associated with higher likelihood of psychotic experiences. In contrast, lower childhood nonverbal ability and greater nonverbal developmental lag were associated with case-level emotional symptoms. Of particular interest, greater verbal–nonverbal discrepancy towards lower nonverbal function is associated with cortical thinning in the rostral part of the prefrontal cortex [32], one of the regions responsible for executive functions as well as major depression.

Finally, although there is little question that fluid cognitive functions typically decline with age, it is less clear how emotional symptoms change over this stage of the life course. A popular view is that they show age-associated decline rather than rising in correspondence with cognitive decline. This is the familiar inverted-U pattern, where symptoms are infrequent during development, rise to a peak in midlife, then decline again. It was originally reported by Blanchflower and Oswald [33] using multiple cross-sectional datasets that are potentially subject to cohort effects; but it was also observed in NSHD [34•]. However, this is not a universal phenomenon, and may depend on the effect of control variables influenced by age as well as the type of statistical modelling [35].

### Social Influences

**Fig. 1:**
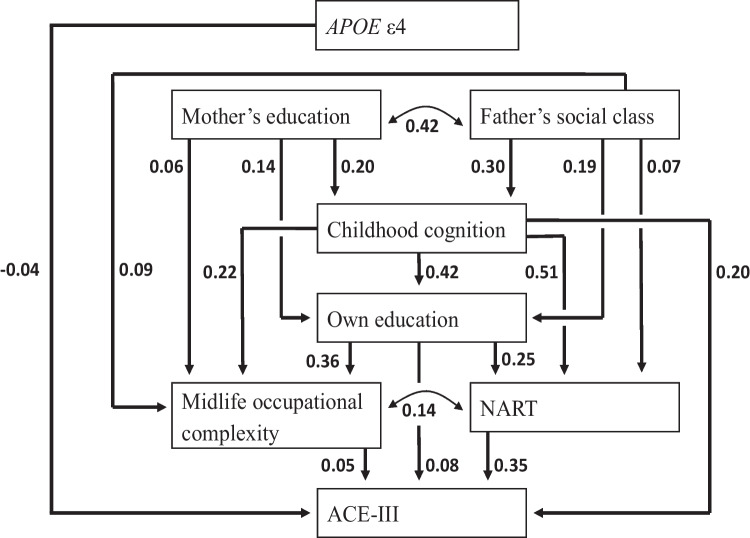
Path model (reproduced from Richards et al. BMJ Open 2019;9:e024404)

Gradients of social inequalities in health are among the most universal phenomena in epidemiology, and the health outcome of cognitive function is no exception. In NSHD, socioeconomic position (SEP) is represented primarily by occupation, of the father in childhood and by own occupation in adulthood, coded according to the UK Registrar General system: professional, intermediate, skilled nonmanual, skilled manual, semi-skilled, and unskilled. Hurst et al. [36] showed that the relative difference between those at the top and bottom of the childhood SEP distribution was as much as 20% for verbal memory in late middle age. This inequality was attenuated but still persistent after adjustment for adult SEP. The childhood SEP gradient was largely explained by childhood cognition and education, the importance of which can be seen in Fig. 1. A different impression emerged in this cohort, however, when a measure of crystallised verbal ability provided the outcome, in this case, a test of pronunciation of irregular words (National Adult Reading Test; NART). For this outcome, Landy et al. [37] found stronger effects for early and later adult SEP than for childhood SEP. To some extent, this is echoed in an earlier version of the Richards et al. path model [38], which included the NART as an outcome.

Of note, Landy et al. used educational attainment rather than occupation to represent early adult SEP. While most social theorists regard education as a different conceptualisation of SEP, this leads to consideration of education itself as an influence on cognitive ageing. This has been a controversial issue, due to the view in some quarters that education is little more than a proxy for intelligence. ‘Entity’ theorists have also argued that the latter is fixed in childhood, in contrast to ‘incremental’ theorists, who maintain that it can be augmented. Space precludes a detailed historical review of this issue, but the argument played out in NSHD [39]. It is clear from Fig. 1 that education shows a strong path to cognitive state net of childhood cognition, largely mediated by the NART. The independent association between education and later cognition was also seen in NCDS and WLS [40]. This effect on later cognition shows a dose–response. NSHD participants were asked at age 36 years if they undertook any training courses, spare-time evening classes, or obtained any higher or further education in the past 5 years. At 43 years, they were asked if they had undertaken any educational or work-related training or taken any examinations since their previous interview. At both ages, adult education was grouped into four categories: none; some but with no resulting qualification; some with a resulting basic qualification; and some with a resulting advanced qualification. After controlling for childhood cognition, formal education, and adult occupational mobility, adult education was positively associated with midlife verbal ability, memory, and fluency [41]. Finally, this appears to show a cohort effect. A stronger association between education and the proxies of everyday literacy and numeracy was found in NCDS than in NSHD [42]. Of note, the minimal school leaving age was raised from 15 to 16 years during the years between these cohorts, resulting in a substantial rise in the number of participants in NCDS leaving school with qualifications.

### Biological Influences

#### Genetic and Epigenetic

Genome-wide association studies (GWAS) have shown that a substantial proportion of individual variation in general cognitive ability is genetic [43]. The best-known genetic influence on dementia — sporadic Alzheimer’s disease (AD) in particular — is the ε4 allele of the apolipoprotein E (*APOE*) gene, which is located on chromosome 17 and is involved in lipid transport (for a recent review, see [44]). This allele appears to have a long occult phase. In Fig. 1, ε4 shows a subtle and direct influence on cognitive state in NSHD, independent of parental SEP, childhood cognition, education, and midlife occupational complexity. This was followed up in more detail by Rawle et al. [45], who showed faster decline in verbal memory from ages 43 to 69 in homozygotes, controlling for sex and childhood cognition. Regarding mechanism, Insight 46 shows that ε4 is specifically associated with β-amyloid peptide (Aβ) [46], a key driver of the AD neurodegenerative process. This is a protein found in the fatty membrane surrounding nerve cells. It is chemically ‘sticky’, gradually clumping together and interfering with nerve function and triggering inflammation. These clumps build into plaques, one of the hallmark microscopic features of AD. However, the role of ε4 appears to be complex, with evidence of selective advantage as well. For example, further work in Insight 46 found that APOE4 and Aβ had opposing effects on visual working memory, predicting better and poorer recall respectively [47]. This may be due to antagonistic pleiotropy, where a gene has positive and negative effects, with the negative effects generally manifesting in later life when the pressure of natural selection is weaker [48]. It may explain why ε4 persists in human populations rather than being replaced by the more benign ε3 and ε2 alleles [47].

It is now well-established that gene expression can be modified, referred to as epigenetic alteration. The classic demonstration comes from studies of offspring of high-nurturing rats, which show higher levels of glucocorticoid receptor gene expression in the hippocampus [49], with implications for cognition. Genomic imprinting involves an inherited chemical change to a DNA sequence. This modification, which is passed from parent to offspring, changes the function of the gene or gene product. Using the Aberdeen birth cohorts, Lorgen-Ritchie et al. [50, 51] showed that imprint methylation (where methyl groups attach to certain DNA segments) of various genes was associated with hippocampal volume and childhood and later-life cognition, suggesting a mechanism though which the complex phenotype of cognition can be inherited. Maddock et al. [52] showed that older biological age in NSHD, as measured by two DNA methylation–based age biomarkers of ageing (PhenoAge (or Levine clock) and GrimAge) at 53, were associated with lower verbal memory and poorer letter search at age 53 in NSHD, but no difference in rate of decline in these measures from 53 to 69, and no association between DNA methylation age at age 43 and cognition at 50 in NCDS.

#### Cardiovascular Function

A range of systemic functions have been investigated in relation to cognition in the birth cohorts. In NSHD, these include lung function [53], kidney function [54], and bone mineral density [55]. However, cardiac and cardiometabolic function have been more extensively studied in this context. In their NSHD-based study, Masi et al. [31] tested associations between cognition and carotid-to-femoral pulse wave velocity (PWV) and common carotid artery intima-media thickness (cIMT) at age 60–64, which are validated surrogate markers of arterial stiffness and atherosclerotic cardiovascular disease. Higher PWV and cIMT were associated with lower verbal memory after adjustment for sex, education, childhood cognition, SEP, systolic blood pressure (SBP), and heart rate. However, these associations were explained by further adjustment for total cholesterol, smoking, diabetes, and levels of physical activity. No associations were observed for timed letter search or reaction time, and no associations were observed between cIMT and any cognitive measure.

In the Lothian 1936 cohort, on average blood pressure rose in the first waves and subsequently fell. However, while childhood cognition was associated with lower blood pressure, intercepts, and trajectories of the latter were not associated with subsequent cognition [56]. Similarly, antihypertensive medication was associated with lower blood pressure, but not with better cognition [56]. After follow-up to age 92 years and using linkage to electronic health records, 410 participants in this cohort remained dementia-free, and 110 had developed probable dementia. Of 234 with complete data (48 with dementia), *APOE* ε4 and greater lifetime physical activity were associated with increased risk of dementia whereas the inverse was found for hypertension [57]. The latter finding is notable and consistent with a phenomenon long recognised, that hypotension in old age is a risk factor for dementia [58]. At the mechanistic level, cerebral small vessel disease at age 73 in this cohort was associated with decline in general cognitive ability, processing speed, verbal memory, and visuospatial ability from ages 73 to 82, controlling for age, sex, vascular risk, and childhood cognitive ability [59].

Insight 46 has shed valuable light on mechanisms behind links between blood pressure and brain changes. In this study, higher SBP and diastolic blood pressure (DBP) at age 53 years and greater increase in SBP and DBP between 43 and 53 years, were associated with higher white matter hyperintensity volume at age 69–71. Higher DBP at 43 years, and greater increase in DBP between 36 and 43, were associated with smaller whole-brain volume. Greater increase in SBP between 36 and 43 was also associated with smaller hippocampal volume [46]. These authors reported similar findings using the Framingham Heart Study-cardiovascular risk score (FHS-CVS) [60••]. Notably, in the first of these studies, neither absolute blood pressure nor change in blood pressure predicted cognition. However, at the time of writing, Insight 46 is collecting data for its third follow-up, after which this issue will be revisited.

## Conclusions

Birth cohorts provide powerful designs for investigating life course influences on cognitive ageing, cognitive impairment, and risk of dementia. For many of these cohorts, information is collected prospectively, before outcomes are known; but even when data are recovered from historical records, typically this information is objective in relation to the aims of the cohort study. Of course, there are limitations: as with almost all longitudinal studies, birth cohorts are vulnerable to attrition of those socially disadvantaged and less healthy, although to some extent this can be mitigated by statistical modelling of missing data. Much information is self-reported, including the basics of sociodemographic and socioeconomic status, not all of which is possible to validate. And there are numerous challenges to the continuity of data, from the necessity of age-appropriate measures, to changing scientific priorities and verities, not all of which are possible to foresee. Nevertheless, with a span of over 100 years, birth cohorts have been yielding information that is crucial to understanding how our minds and brains age, with the hope of mitigating some of the most devastating and costly conditions of old age.
